# Highly Efficient Mesoporous Carbonaceous CeO_2_ Catalyst for Dephosphorylation

**DOI:** 10.1021/acsomega.2c01832

**Published:** 2022-06-17

**Authors:** Aashima Sharma, Surinder K. Mehta, Avtar S. Matharu

**Affiliations:** † Green Chemistry Centre of Excellence, Department of Chemistry, University of York, York YO10 5DD, England; ‡ Department of Chemistry and Centre for Advanced Studies in Chemistry, Panjab University, Chandigarh 160014, India

## Abstract

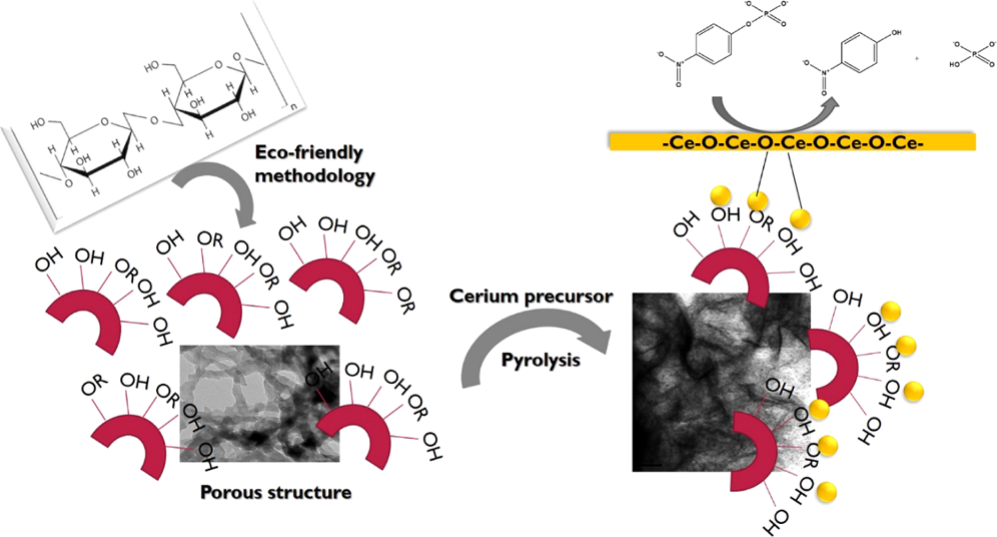

Phosphorus is fast becoming a critical element, as the global supply and demand are reaching unsustainable levels. Herein, the synthesis, characterization, and applicability of a novel biomass-derived mesoporous carbonaceous material decorated with CeO_2_ (CeO_2_-S400) as an efficient catalyst for the dephosphorylation of 4-nitrophenyl phosphate disodium salt hexahydrate are reported. The presence and distribution of CeO_2_ are evidenced by inductively coupled plasma mass spectrometry (ICP-MS) (118.7 mg/g), high-resolution transmission electron microscopy (HRTEM), and energy dispersive X-ray (EDX) mapping. The apparent rate constant for the efficient catalysis of 4-nitrophenyl phosphate disodium salt hexahydrate was 0.097 ± 0.01 for CeO_2_-ES and 0.15 ± 0.03 min^–1^ for CeO_2_-S400, which followed first-order kinetics. Rate constants normalized by the catalytic loading (*k*_m_) were 80.84 and 15.00 g^–1^ min^–1^ for CeO_2_-ES and CeO_2_-S400, respectively, and the normalized rate constants with respect to surface area were 3.38 and 0.04 m^–2^ min^–1^ for CeO_2_-ES and CeO_2_-S400, respectively. This indicates that the presence of CeO_2_ nanoparticles has a catalytic effect on the dephosphorylation reaction.

## 1 Introduction

As the global population continues to increase, food supply and food security have become a grand challenge, as does the importance of phosphorus because of its use in fertilizers.^1−3^ However, phosphorus is fast becoming a critical element in many parts of the world, as supply cannot meet demand. Phosphorus is an important constituent in soil, but its concentration gradient can vary from one geographic region to another. The quality and easy availability of existing phosphate rocks are declining.^4^ The production of phosphate rock is predicted to reach its peak before 2040, and the reserves will be wholly exhausted by the end of this century.^5^ The extraction of phosphorus is an expensive task, and the catalytic performance of natural phosphatases is sensitive to the environmental conditions, for example, high reaction temperature, pH, etc.^6^ Therefore, synthetic catalysts that are alternatives of natural phosphatases are emerging as promising candidates because of their stability and cost effectiveness. One interesting way forward to obtain phosphorus is from already phosphorylated biomolecules^7^ via catalytic dephosphorylation, which hydrolytically cleaves phosphate ester bonds to release free phosphate anions that can be reutilized, for example, in fertilizer production.^8,9^

In last few years, nanoceria (CeO_2_) has been reported to exhibit multiple enzymatic activities, including superoxidase and catalase because of its ability to switch between +3 and +4 oxidation states.^10^ CeO_2_ is ideal for dephosphorylation because the oxygen vacancies within its structure are believed to be active sites for catalytic dephosphorylation.^11^ Manto et al.^12^ reported dephosphorylation for phosphorus recovery from organic and biological molecules using CeO_2_ with different morphologies. Kuchma et al.^13^ investigated the dephosphorylation activity of CeO_2_ with respect to the presence of Ce^3+^ and Ce^4+^ sites, concluding that the latter inhibited catalytic activity. However, the use of unbound or homogeneous nano-CeO_2_ is problematic because of leaching of Ce^3+^/Ce^4+^ ions into solvent media and/or binding to substrates. Leaching, size, and agglomeration of CeO_2_ can be prevented via depositing CeO_2_ on a porous solid support, thus not affecting its reactivity.^14^ Therefore, mesoporous carbonaceous materials derived from polysaccharides (Starbons) may prove to be ideal substrate materials due to their tunable functionality and surface composition.^15^ Polysaccharides, such as starch and alginic acid, can be employed as precursors for the manufacture of carbonaceous materials with multiple porosities ranging from micro (<2 nm) to meso (>2 and <50 nm) to macro (>50 nm).^16^ Starbons have been widely employed for environmental remediation, for example, dye and metal adsorption,^17^ but their utility as a support in catalysis for the dephosphorylation reaction is novel.

Thus, this research explores the synthesis and characterization of a novel mesoporous material impregnated with CeO_2_, derived from noncarbonized expanded starch (CeO_2_-ES), and its corresponding carbonized equivalent (CeO_2_-S400). The usefulness as a dephosphorylation catalyst is explored for the conversion of 4-nitrophenyl phosphate disodium salt hexahydrate in the aqueous phase to 4-nitrophenol. 4-Nitrophenyl phosphate disodium is a model compound used for the dephosphorylation reaction because its conversion can be easily tracked by UV–visible spectroscopy. The importance of the prepared structures is an environmentally friendly biomass-based support system for CeO_2_ nanoparticles and their uniform distribution over the highly porous Starbon bed and for CeO_2_ to act as active sites for the dephosphorylation catalytic reactions. The dephosphorylation catalytic efficiency of the materials will be investigated along with their reusability.

## 2 Experimental Section

### 2.1 Reagents

All the chemicals were reagent grade and used without any further purification. Hylon VII high-amylose corn starch (HACS, 75% amylose content) was purchased from National Starch and Chemical limited. Cerium acetate (81–83%), *para*-toluene sulfonic acid (PTSA) (≥98.5%), *tert*-butanol (TBA, ≥99.0%), sodium hydroxide, *para*-nitrophenyl phosphate disodium salt hexahydrate (*p*-NPP, ≥99%), l-ascorbic acid, ammonium molybdate, and sulfuric acid were purchased from Sigma-Aldrich Ltd. Absolute ethanol and acetone were obtained from VWR Chemicals. Deionized water was supplied in the laboratory via an ELGA Centra system.

### 2.2 Microwave and Carbonizing Process

In the first step, the expansion of Hylon VII was carried out using a CEM Mars 6 Microwave reactor. The carbonization process was performed in a muffle furnace with the following protocol: first stage: temperature increased from ambient to 100 °C at a rate of 5 °C min^–1^; second stage: temperature increased to 210 °C at a rate of 0.2 °C min^–1^; third stage: temperature increased to 400 °C min^–1^ and held for 60 min.

### 2.3 Dephosphorylation Catalytic Studies

A stock solution of 4-nitrophenyl phosphate disodium salt hexahydrate (*p*-NPP) was first prepared by dissolving *p*-NPP (20 mg) in ethanol (100 mL). An aliquot of stock solution (10 mL) was taken with varying amounts of synthesized systems, and then, the solution was heated to the desired reaction temperature. As the reaction proceeded, the solutions turned from turbid white to turbid yellow, indicating the formation of *para*-nitrophenol (*p*-NP). At different time intervals, 1 mL of the reaction was collected, and then, the solution was centrifuged at a speed of 16 000 rpm for 10 min, and UV–visible spectra were recorded of the collected solution.

### 2.4 Molybdenum Blue Assay

Aqueous 0.1 M l-ascorbic acid (10 mL) was added to a freshly prepared mixture of ammonium molybdate solution (5 mL, 4 wt % in water) and aqueous 5.0 N sulfuric acid (17 mL) and gently stirred at room temperature. Upon mixing, the solution turned golden yellow.

Stock phosphate solutions were prepared by dissolving Na_2_HPO_4_ (5 mg) in deionized water (50 mL). A series of dilutions were carried out to prepare the phosphate standards. To 1 mL of each standard, 200 μL of the reagent mixture was added, and the solution slowly turned blue. A total of 200 μL of each standard was dispensed to a microplate for ultraviolet–visible (UV–vis) spectroscopy analysis at 890 nm, and a calibration curve for the phosphate concentration was constructed. To each 1 mL supernatant extracted during the model dephosphorylation reactions, 200 μL of the reagent mixture was added. The supernatants quickly changed color from yellow to clear to blue and were analyzed via UV–vis at 890 nm to quantify the amount of the phosphate present.

### 2.5 Synthesis of CeO_2_ Nanoparticles Decorated on Starbon (CeO_2_-S400)

HACS and water were mixed in a ratio of 1:10 (w/v) to form a homogeneous mixture.^18^ The mixture was poured into a Teflon vessel and microwaved at 140 °C for 10 min at 800 psi and 800 W. Thereafter, the mixture was retrograded (4 °C for 48 h), and the final product was labeled as pure expanded starch (ES). The latter was further macerated, PTSA and *tert*-butanol were added, and the mixture was stirred at room temperature overnight followed by freeze drying to afford a white-colored flaky solid. This material was carbonized under vacuum at 400 °C to yield a black powder (54%), which was labeled as S-400.

The above-mentioned synthetic route was adapted to afford CeO_2_-S400 as follows. After the retrogradation, cerium acetate (5 w %) with 1 mM sodium hydroxide was added, and the mixture was stirred overnight. The resultant, expanded, pale-yellow solid, indicative of the presence of ceria, was labeled as CeO_2_-ES (Figure S1) and was subsequently pyrolyzed at 400 °C to afford the desired CeO_2_-S400 (57%).

## 3 Results and Discussion

### 3.1 Infrared Analysis

**Figure 1:**
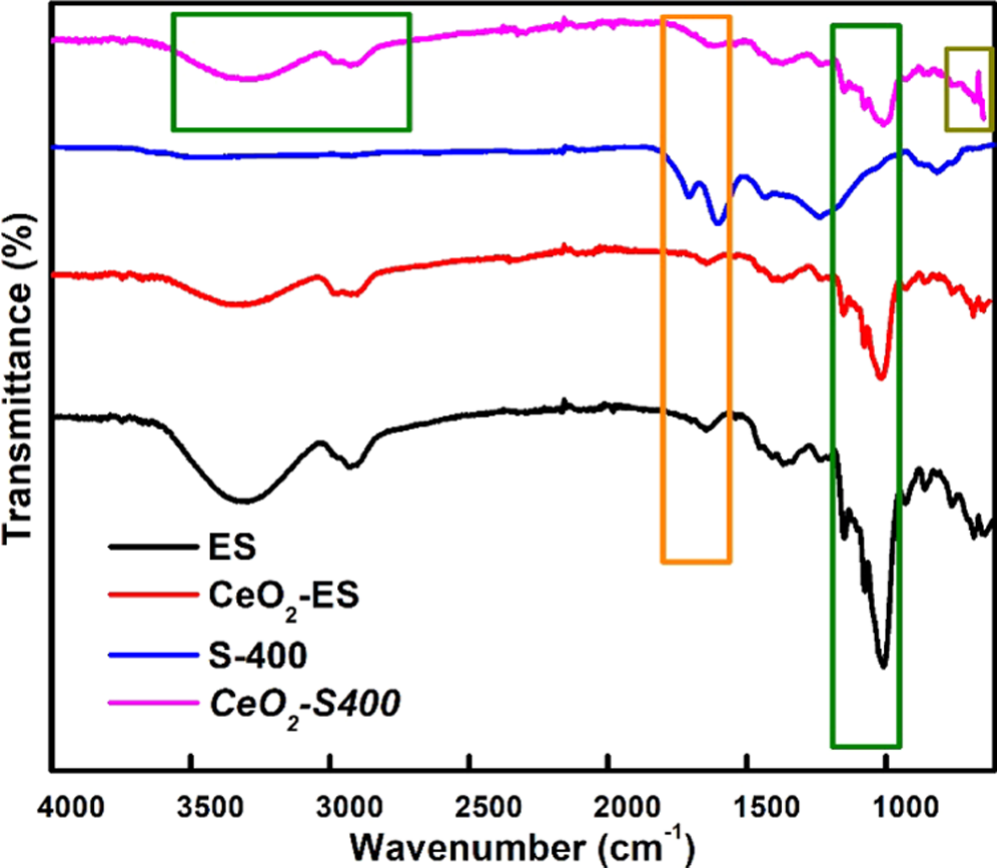
IR spectra for expanded starch (ES), cerium oxide-impregnated expanded starch (CeO_2_-ES), carbonized expanded starch (S400), and cerium oxide-impregnated carbonized starch (CeO_2_-S400).

The FTIR analysis (Figure 1) of the synthesized systems shows a weak, broad absorbance band centered at around 3300 cm^–1^ indicative of the O–H stretching frequency and a strong absorbance band at 1009 cm^–1^ due to the C–O stretching vibration, which decreases on pyrolysis.^19^ In the case of CeO_2_-S400, the absorbance band at around 1014 cm^–1^ may be due to the CO_3_^2–^ bending vibration and C–O stretching vibration, which may get trapped during the synthesis procedure. The absorbance band at 1655 cm^–1^ is synonymous with C=C stretching vibrations,^20^ which indicates the existence of aromatic nature in the synthesized systems. The band at around 652 cm^–1^ is ascribed to the Ce–O stretching frequency.^21^

### 3.2 Thermogravimetric Analysis

**Figure 2:**
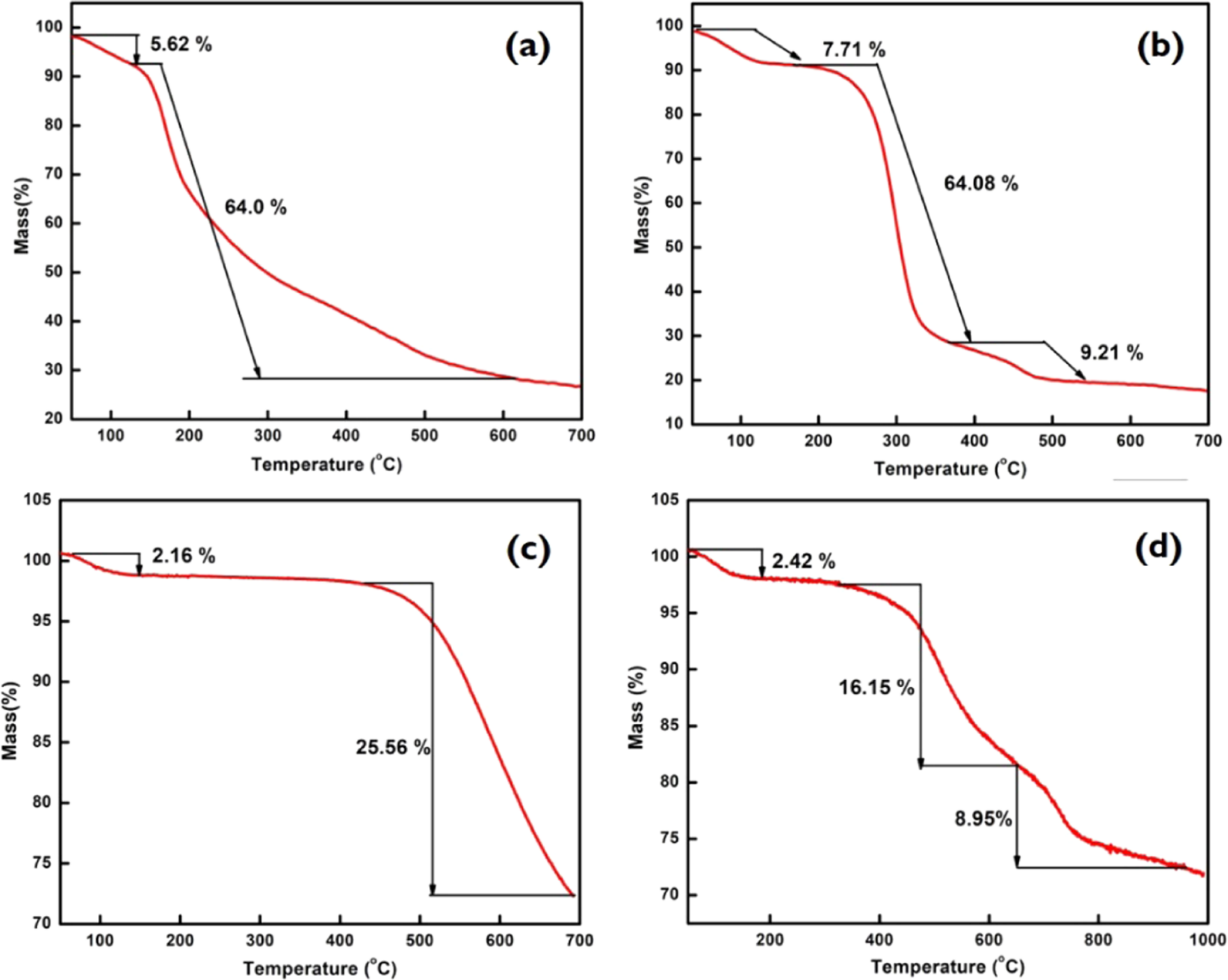
Thermogravimetric analysis of (a) expanded starch (ES), (b) cerium oxide-impregnated expanded starch (CeO_2_-ES), (c) carbonized expanded starch (S400), and (d) cerium oxide-impregnated carbonized starch (CeO_2_-S400).

The thermal properties (TGA) on converting expanded starch (ES) into CeO_2_-ES, S-400, and CeO_2_-S400 are displayed in Figure 2. In all cases, the first mass loss, which occurs from room temperature to 150 °C, is attributed to the loss of water and any residual volatiles.^22^ The TGA of expanded starch (Figure 2a) reveals multiple decomposition events from 150 to 550 °C synonymous with degradation of the polysaccharide chain and affords about 27% of residue.^23^ The TGA of CeO_2_-ES (Figure 2b) is much better resolved and displays increased thermal stability of the polysaccharide chain from ∼190 °C (Figure 2a) to ∼210 °C (Figure 2b). Rapid decomposition of expanded starch is noted from 210 to 300 °C, which may be associated with noncomplexed or nonbound ceria or ceria that selectively binds with amylose and amylopectin chains. Interestingly, a very distinct decomposition region is now also observed from 300 to 400 °C accounting for 9.21% of the total mass loss.

The successful carbonization of expanded starch (ES) to S-400 (Figure 2c) is evidenced by the presence of a flat line in the region of 150–450 °C followed by the onset of a major decomposition at 500–480 °C. In the case of CeO_2_-S400 (Figure 2d), the first weight loss was about 2.5% due to the loss of water around 100 °C. The second step leads to a maximum weight loss of 16% due to the decomposition of the intercalated structure of Starbon.^23^ The weight loss (8.95%) at around 600 °C is due to the loss of oxygen at high temperatures from CeO_2_.^24^ The incorporation of CeO_2_ into the matrix of the carbonaceous material changes the degradation pathway.^25^

### 3.3 N_2_ Adsorption Porosimetry

The nitrogen adsorption–desorption isotherms of the prepared samples are shown in Figure S3 and are classified as type IV (IUPAC), and their porosity data are summarized in Table 1. In the case of ES and CeO_2_-ES (Figure S3a,b), the desorption curves showed the forced closure phenomenon, as the closure point changed from 0.4 to 0.5 relative pressure. This can be attributed to the instability of the meniscus condensation for pores around 4 nm.^26^

**Table 1:** Nitrogen Porosimetry Data for Expanded Starch (ES), Cerium Oxide-Impregnated Expanded Starch (CeO_2_-ES), Carbonized Expanded Starch (S400), and Cerium Oxide-Impregnated Carbonized Starch (CeO_2_-S400)

sample	BET surface area (m^2^/g)	mesopore volume/micropore volume at *p*/*p*_0_ = 0.90 (%)	micropore volume (cm^3^/g)	mesopore volume (cm^3^/g)	average pore diameter (nm)
ES	26	84.8	0.0014	0.0334	5.85
CeO_2_-ES	24	90.3	0.0034	0.0318	5.42
S-400	667	37.3	0.2471	0.1470	2.32
CeO_2_-S400	345	20.2	0.1287	0.0325	1.84

For S-400 and CeO_2_-S400, the hysteresis loop did not close under low pressure, which may be due to deformation as a result of the soft nature of the material (Figure S3c,d) or trapped nitrogen that cannot be released.^27^ The observed surface area of both ES (26 m^2^/g) and CeO_2_-ES (24 m^2^/g) significantly increased on carbonization, S-400 (667 m^2^/g) and CeO_2_-S400 (345 m^2^/g), respectively. The decreases observed in the surface areas of the Ce-containing materials with respect to the original solids (ES vs CeO_2_-ES and S-400 vs CeO_2_-S400) may account for the accumulation of nanoparticles either in the surface or in pores, i.e., blocking of pores. The total micropore volume was found to be 0.247 and 0.128 cm^3^/g for S-400 and CeO_2_-S400, respectively. The contribution of mesoporosity with respect to the total pore volume decreased upon carbonization, as did the pore volume. A decrease in the pore volume may also be due to the formation/inclusion of nanoparticles within pores.

### 3.4 Scanning Electron Microscopy (SEM)

**Figure 3:**
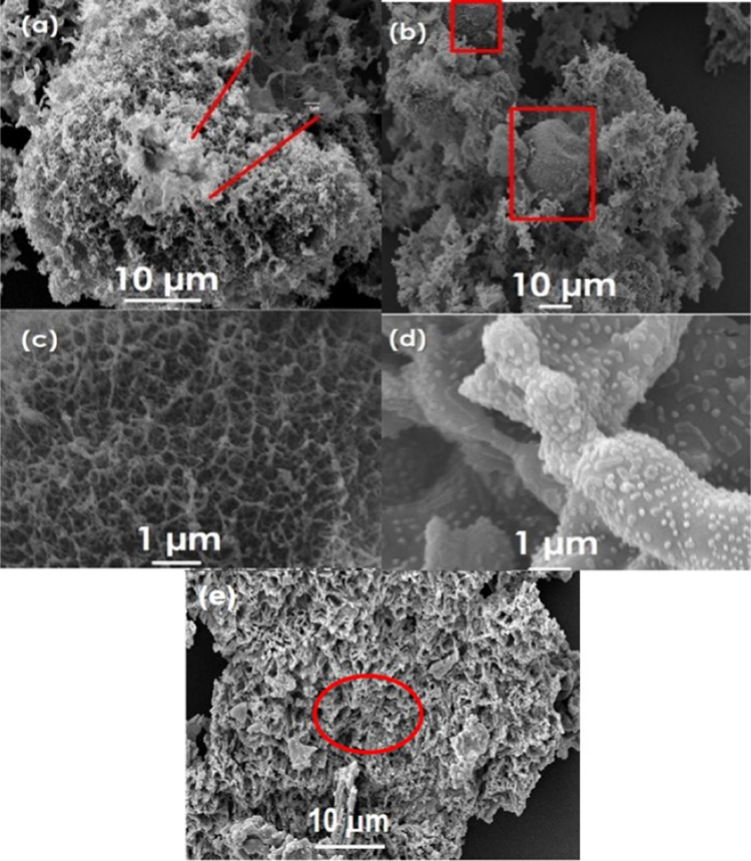
SEM images of (a) ES, (b) CeO_2_-ES, (c) Starbon@400, (d) CeO_2_-S400 at 1 μm magnification, and (e) CeO_2_-S400 at 10 μm magnification. The marked zones depict the following characteristics of the materials: In (a), the zoom-out image of one portion of ES represents the interconnected network present throughout the material. In (b), the highlighted portion indicates the presence of globules, which depicts the presence of nanoparticles along with the parent network. In (e), the image has been taken at 10 μm to visualize the presence of the network in the pyrolyzed product also.

The SEM images (Figure 3) confirm the presence of porosity within the synthesized materials with/without the presence of nanoparticles. Figure 3a shows the porous network structure of pure ES, and CeO_2_-ES (Figure 3b) represents globules along with the parent network, which depicts the presence of nanoparticles, which results during the drying of the xerogel. The interconnected networking can be seen in all the synthesized systems, reflecting porosity. The porosity of the material was maintained after the incorporation of the cerium precursor, which can be seen in Figure 3c,d.

The basic morphology remains unaltered, but the presence of clumps in between the interconnected structure can be seen, which indicates the presence of nanoparticles. After pyrolysis, CeO_2_-S400 showed the presence of spherical particles, indicative of the presence of CeO_2_ nanoparticles (Figure 3d). The homogeneous distribution of the nanoparticles over the interconnected network was also assessed by EDX mapping (Figure S4), which confirms that the nanoparticles are not concentrated in one part but finely distributed over the entire network.

### 3.5 Transmission Electron Microscopy (TEM) and HRTEM

**Figure 4:**
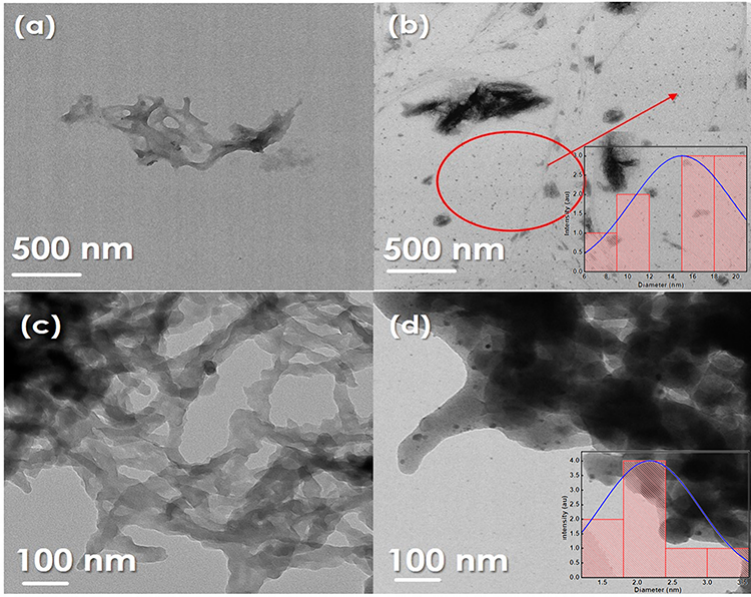
TEM images of (a) ES, (b) CeO_2_ -ES, (c) Starbon@400, and (d) CeO_2_-S400.

**Figure 5:**
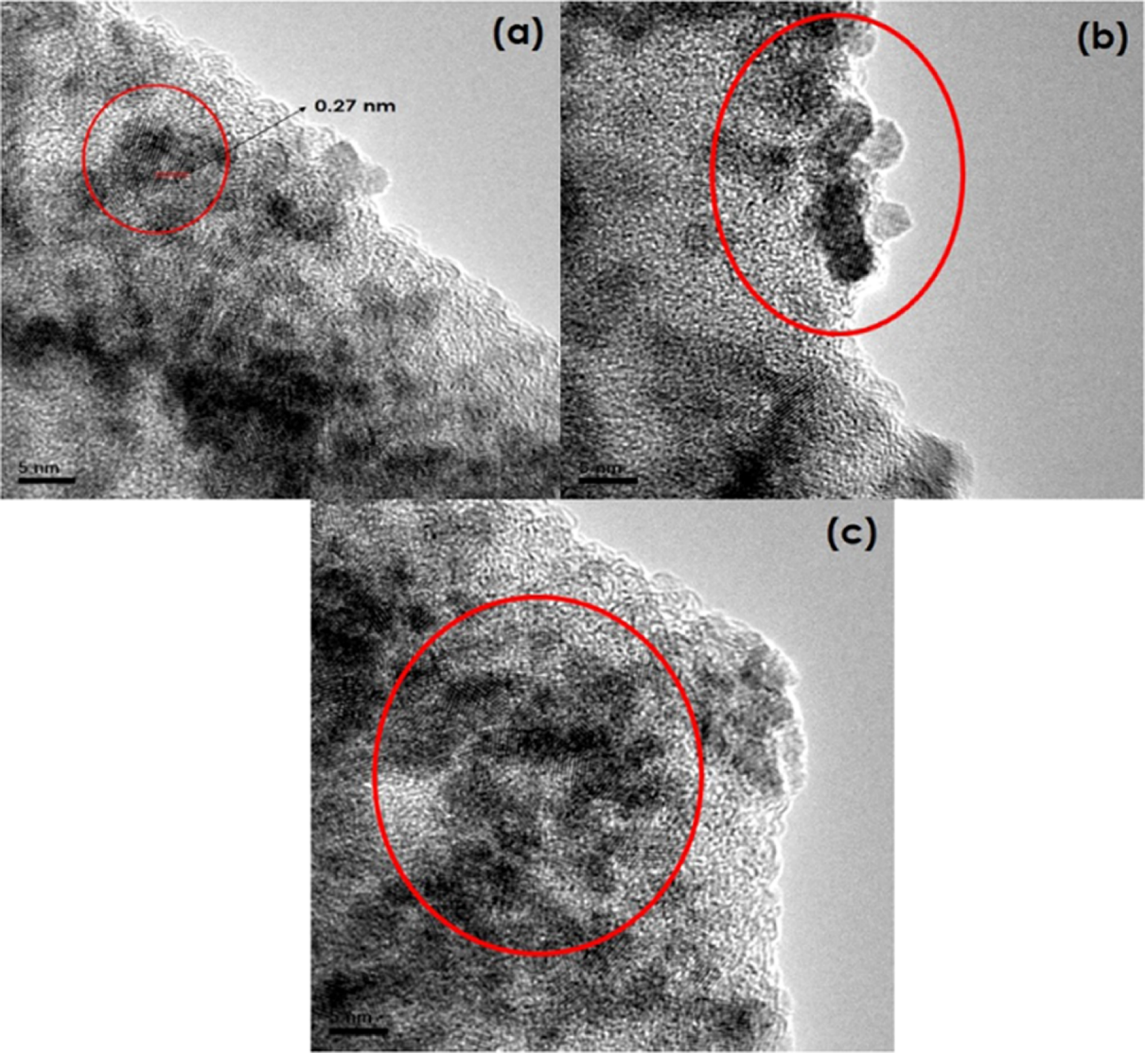
HRTEM images of (a) CeO_2_-ES and (b, c) CeO_2_-S400.

TEM images (Figure 4a,b) show the presence of a homogeneous structure around the pores in the case of expanded starch (ES) and CeO_2_-decorated expanded starch (CeO_2_-ES). The formation of an intercalated structure was observed (Figure 4c) due to the removal of the entrapped gases/products as the material tends toward a sp^2^ carbon structure. The process of carbonization initiates around the mesopores because the acid is absorbed on the outer surface and pores. Figure 4d indicates the presence of an intercalated structure and nanoparticles, which are spread over the carbonaceous material. The size of the nanoparticles was calculated using J image to afford sizes of 14 and 2.5 nm for CeO_2_-ES and CeO_2_-S400, respectively (see the inset of Figure 4). On closer inspection, HRTEM (Figure 5) clearly showed the surface and pores to be decorated with spherical-shaped nanoparticles of around 7 nm diameter possessing a lattice fringe with a d-spacing of 0.27 nm corresponding to the (111) facet of the FCC of CeO_2_ nanoparticles.

### 3.6 X-ray Photoelectron Spectroscopy

**Figure 6:**
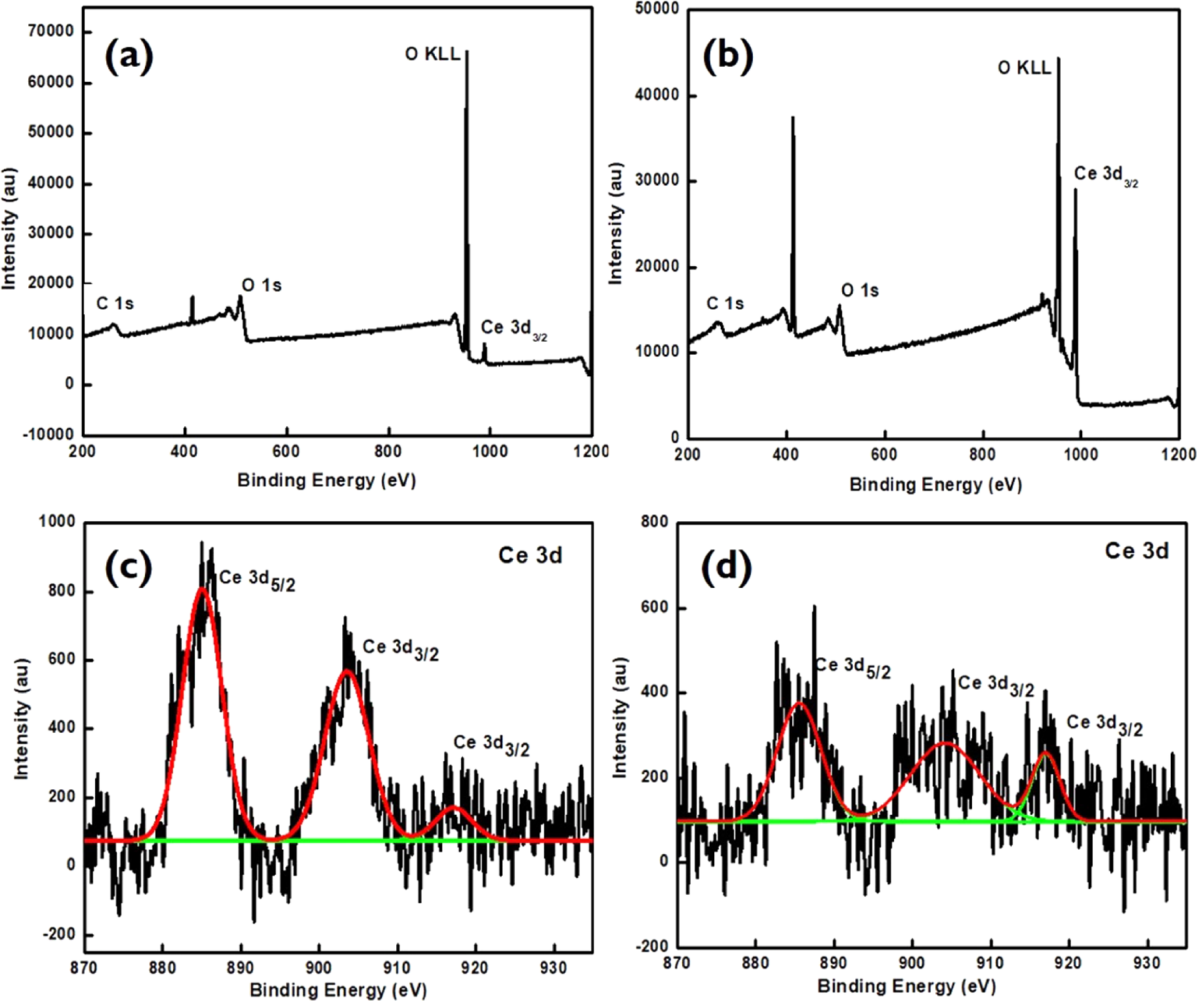
XPS survey of (a) CeO_2_-ES, (b) CeO_2_-S400, and Ce-edge of (c) CeO_2_-ES and (d) CeO_2_-S400.

The nature and binding of cerium oxide nanoparticles were investigated by XPS. Figure 6a,b represents the XPS survey of CeO_2_-ES and CeO_2_-S400, which indicated the presence of carbon, oxygen, and cerium in the systems. Figure 6c,d depicts the characteristic peak of the Ce 3d shell and confirms the Ce^3+^ and Ce^4+^ states. The peaks observed at 885.27 and 903.47 eV are due to the spin–orbit coupling of the 3d_5/2_ and 3d_3/2_ levels, respectively. In detail, the peak located at 916.78 eV is ascribed to the 3d^10^4f^1^ electronic state of Ce^4+^, whereas the peaks positioned at 885.27 and 903.47 eV are attributed to the 3d^10^4f^1^ states of Ce^3+^.^28,29^ The oxygen edge of the synthesized samples showed peaks at 531.72 and 533.26 eV, which are due to C=O and C–O, respectively.^30^ A peak at 538.34 eV in the oxygen edge is chemically bound oxygen to the lattice and chemisorbed oxygen.^31^ The deconvoluted spectra of the carbon edge showed binding energy peaks at 284.49 and 284.94 eV representing C sp^2^ and C sp^3^, respectively. The peaks at 288.93, 286.45, and 291.20 eV present O–C=O, C–O, and Π–Π* transitions, respectively.^32^

### 3.7 Catalytic Studies and Dephosphorylation Kinetic Evaluation

**Figure 7:**
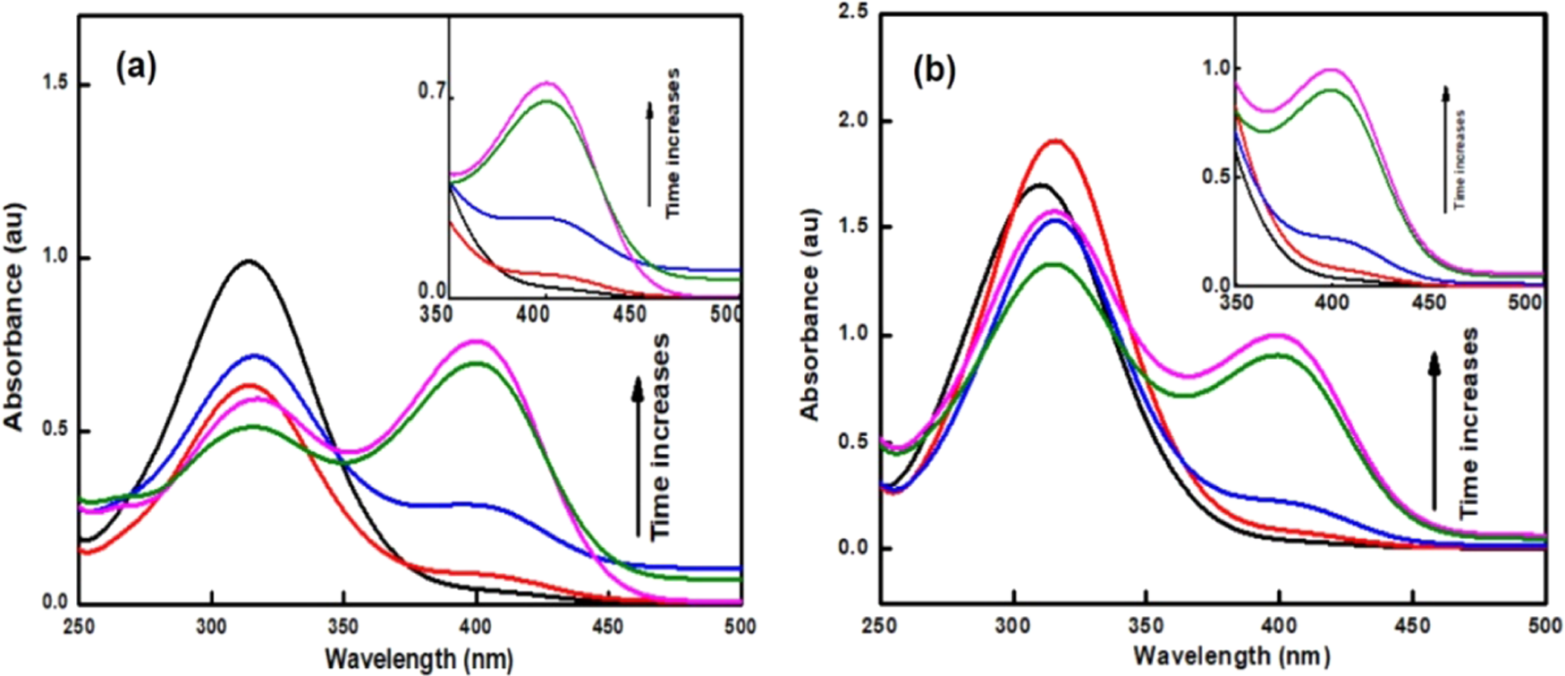
UV spectra monitoring the progress of the catalytic reaction over the time course of (a) CeO_2_-ES and (b) CeO_2_-S400.

The prepared systems were investigated for their catalytic ability to dephosphorylate waste *p*-NPP (Figure S5) in aqueous solution. *p*-NPP is a common chromogenic substrate used for spectrophotometric analysis of phosphates.^33^ Catalytic cleavage of the phosphate ester bond in *p*-NPP generates free phosphate anion groups and *p*-NP in aqueous solution. The *p*-NPP evidences an absorption peak at around 310 nm, and its hydrolysis product displays a characteristic absorption peak centered around 400 nm in the UV–visible spectrum. Figure 7 shows the UV–vis spectra collected over the course of the dephosphorylation of *p*-NPP using CeO_2_-ES (Figure 7a) and CeO_2_-S400 (Figure 7b) at 40 °C. In the presence of a catalyst, the characteristic absorbance peak of *p*-NP at 405 nm after 3 h was enhanced significantly. The presence of Ce^3+^ at the surface and matrix is crucial for the catalytic reaction, which is responsible for cleavage of the phosphoester bond.

The prepared samples were tested and showed substantially different catalytic activities toward the dephosphorylation reaction (Figure S7). The CeO_2_-loaded samples showed reasonably effective catalysis activity and were pursued for further studies. The apparent rate constant was found to be 0.097 ± 0.01 min^–1^ for CeO_2_-ES and 0.15 ± 0.03 min^–1^ for CeO_2_-S400 and followed first-order kinetics, as depicted in Figure S8. Rate constants normalized by the catalytic loading (*k*_m_) were 80.84 and 15.00 g^–1^ min^–1^ for CeO_2_-ES and CeO_2_-S400, respectively, and the normalized rate constants with respect to surface area (*k*_s_) were 3.38 and 0.04 m^–2^ min^–1^ for CeO_2_-ES and CeO_2_-S400, respectively. This indicates that the presence of CeO_2_ nanoparticles, which have different ratios of Ce^3+^/Ce^4+^ due to pyrolysis, results in variation in catalytic activity on the dephosphorylation reaction. The plausible mechanism of catalytic conversion initiates with the adsorption of *p*-NPP on CeO_2_ via the interaction between P=O and Ce. The Ce^4+^/Ce^3+^cations coordinate with phosphoryl oxygen and activate the P-O bond. After the completion of the reaction, the product *p*-NP/phosphate can be readily released from the surface by water solvation once SN_2_ hydrolysis is activated (vide infra).^34^ The difference in the rate constant value of CeO_2_-ES and CeO_2_-S400 may be due to the concentration difference of Ce^4+^ and Ce^3+^ ions in the matrix. The Lewis acidity of Ce^3+^ ions plays an important role in the catalytic activity for dephosphorylation.^13^

The effect of pH on the catalytic dephosphorylation reaction is shown in Figure S9. A higher yield (%) of *p*-NP was observed with increasing pH. In the acidic solution, the catalytic efficiency was much low. The yield (%) of *p*-NP reached close to 20% at pH 3.0, while at pH 7.0, the yield (%) of *p*-NP significantly increased to 67%, indicating pH-dependent catalytic performance. Furthermore, increasing the pH resulted only in a slight enhancement of catalytic performance. Because of the slight difference in catalytic efficiency under neutral and alkaline conditions, dephosphorylation was optimized at a pH of 7.0. The effect of the catalyst dose was investigated by varying the amount of the catalyst (2–12 mg). It was noticed that 12 and 10 mg of the catalyst in the case of CeO_2_-ES and CeO_2_-S400 was effective in catalytic dephosphorylation, respectively (Figure S10).

**Figure 8:**
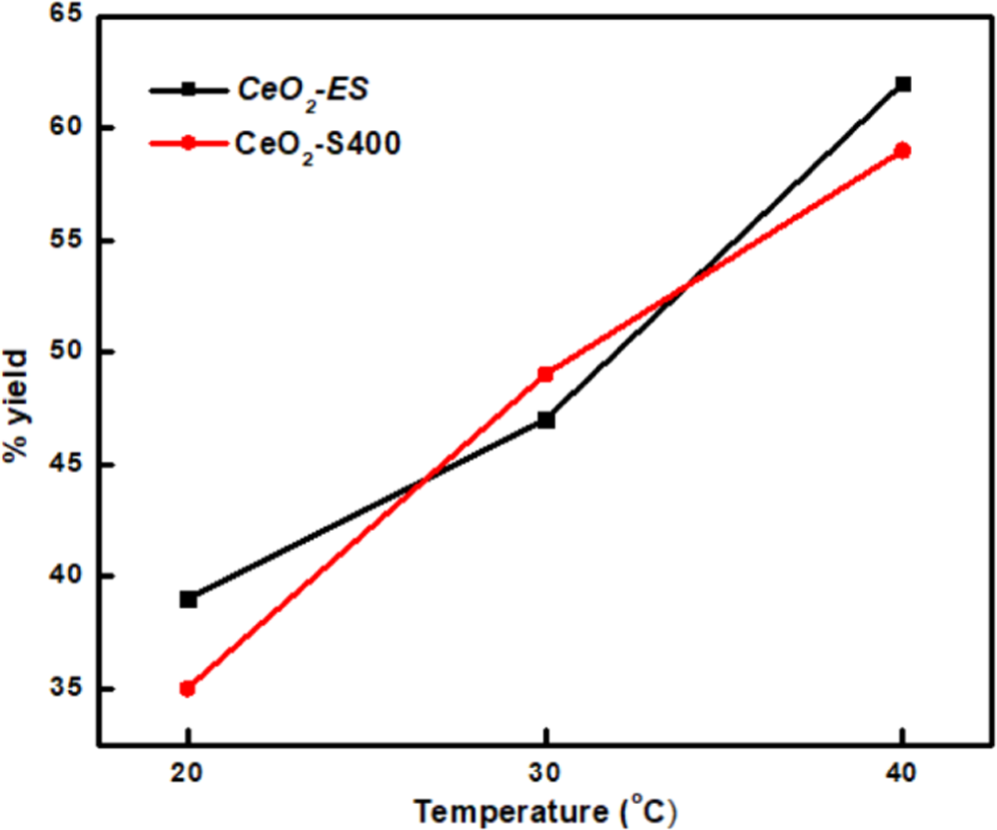
Yield (%) of products formed at various temperatures.

After the catalysis, the catalysts were extracted via centrifugation and scanned to visualize any morphological changes in the catalyst. It was learned that they retain their morphological character with minor changes, as shown in Figure S11. The temperature studies showed an increase in the yield (%) of the reaction (Figure 8). It is a fact that on increasing the temperature, the rate of the reaction increases, and furthermore, more product formation is observed. The surface-adsorbed *p*-NPP molecules that cannot be readily converted to *p*-NP/phosphate may not be recorded, resulting in the observed nonstoichiometric relation between *p*-NPP and *p*-NP. The recyclability experiments depicted that after each cycle, there is a drop in the yield (%) in both of the cases. The drop (65–61%) is more significant in the case of CeO_2_-ES (Figure S12).

## 4 Conclusions

The fabricated materials were manufactured via an environmentally friendly route and derived from a green source, which is important for the future synthesis of biomass-based metal oxide samples. Novel CeO_2_-loaded processed biomass samples were synthesized via the microwave activation method and characterized using various spectroscopic techniques. The samples showed potential as heterogeneous catalysts for the dephosphorylation reaction. The synthesized samples exhibit high porosity and possessed an interconnected pore network, which renders them capable candidates for other adsorption applications. The systematic studies on the model dephosphorylation reaction demonstrated that CeO_2_-loaded samples showed potential as a catalyst for dephosphorylation reactions. The apparent rate constant was found to be 0.97 ± 0.1 min^–1^ for CeO_2_-ES and 0.15 ± 0.3 min^–1^ for CeO_2_-S400, showing a difference in the catalytic performance on processing the starch. Recyclability of the systems represents an important merit of the catalysts in practical applications. Consequently, the present study highlighted the path of these kinds of biomass-based heterogeneous catalysts decorated with CeO_2_ in catalysis applications at a large scale.

## Supporting Information Available

Characterization techniques employed for characterizing the materials and mechanism of the formation of Starbons (PDF)

## References

[ref1] Buckman H. O.; Brady N. C. The Nature and Properties of Soils 6th ed.; Macmillan: New York. 1960.

[ref2] Greenwood N. M.; Earnshaw A. Chemistry of the Elements 2nd ed.; Elsevier: Oxford, 1997.

[ref3] Wong P. Y.; Cheng K. Y.; Kaksonen A. H.; Sutton D. C.; Ginige M. P. A novel post denitrification configuration for phosphorus recovery using polyphosphate accumulating organisms. Water Res. 2013, 47, 6488–6495. 10.1016/j.watres.2013.08.023. 24041527

[ref4] Walan P.; Davidsson S.; Johansson S.; Hook M. Phosphate Rock Production and Depletion: Regional Disaggregate Modelling and Global Implications. J. Resour., Conserv. Recycl. 2014, 93, 178–187. 10.1016/j.resconrec.2014.10.011.

[ref5] Kisinyo P. O.; Asbon O. P. Depletion of phosphate rock reserves and world food crisis: Reality or hoax?. Afr. J. Agric. Res. 2020, 16, 1223–1227. 10.5897/AJAR2020.14892.

[ref6] Lin J. H.; Yang Y. C.; Shih Y. C.; Hung S. Y.; Lu C. Y.; Tseng W. L. Photoinduced electron transfer between Fe(III) and adenosine triphosphate-BODIPY conjugates: Application to alkaline-phosphatase-linked immunoassay. Biosens. Bioelectron. 2016, 77, 242–248. 10.1016/j.bios.2015.09.022. 26409025

[ref7] Tong J.; Chen Y. Enhanced Biological Phosphorus Removal Driven by Short-Chain Fatty Acids Produced from Waste Activated Sludge Alkaline Fermentation. Environ. Sci. Technol. 2007, 41, 7126–7130. 10.1021/es071002n. 17993158

[ref8] Nguyen L. K.; Matallanas D.; Croucher D. R.; Kriegsheim A.; Kholodenko B. N. Signalling by Protein Phosphatases and Drug Development: A Systems-Centred View. FEBS J. 2013, 280, 751–765. 10.1111/j.1742-4658.2012.08522.x. 22340367 PMC3368988

[ref9] Syers K.; Bekunda M.; Cordell D.; Corman J.; Johnston J.; Rosemarin A.; Salcedo I.; Lougheed T. L. United Nations Environment Programme Year Book 2011: Emerging Issues in Our Global Environment In Goverse T.; Bech S., Eds.; United Nations: New York, 2011.

[ref10] Xu C.; Qu X. Cerium oxide nanoparticle: a remarkably versatile rare earth nanomaterial for biological applications. NPG Asia Mater. 2014, 6, e90 10.1038/am.2013.88.

[ref11] Yao T.; Tian Z.; Zhang Y.; Qu Y. Phosphatase-like activity of porous nanorods of CeO_2_ for the highly stabilized dephosphorylation under interferences. ACS Appl. Mater. Interfaces 2019, 11, 195–201. 10.1021/acsami.8b17086. 30556997

[ref12] Manto M. J.; Xie P.; Wang C. Catalytic Dephosphorylation Using Ceria Nanocrystals. ACS Catal. 2017, 7, 1931–1938. 10.1021/acscatal.6b03472.

[ref13] Kuchma M. H.; Komanski C. B.; Colon J.; Teblum A.; Masunov A. E.; Alvarado B.; Babu S.; Seal S.; Summy J.; Baker C. H. Phosphate ester hydrolysis of biologically relevant molecules by cerium oxide nanoparticles. J. Nanomed. Nanotechnol. 2010, 6, 738–744. 10.1016/j.nano.2010.05.004. 20553964

[ref14] Astruc D.; Lu F.; Aranzaes J. R. Nanoparticles as Recyclable Catalysts: The Frontier between Homogeneous and Heterogeneous Catalysis. Angew. Chem., Int. Ed. 2005, 44, 7852–7872. 10.1002/anie.200500766. 16304662

[ref15] Kaur J.; Kaur K.; Mehta S. K.; Matharu A. S. A novel molybdenum oxide–Starbon catalyst for wastewater remediation. J. Mater. Chem. A 2020, 8, 14519–14527. 10.1039/D0TA05388K.

[ref16] Budarin V.; Clark J. H.; Hardy J. J. E.; Luque R.; Milkowski K.; Tavener S. J.; Wilson A. J. Starbons: New Starch-Derived Mesoporous Carbonaceous Materials with Tunable Properties. Angew. Chem., Int. Ed. 2006, 45, 3782–3786. 10.1002/anie.200600460. 16671136

[ref17] Parker H. L.; Hunt A. J.; Budarin V. L.; Shuttleworth P. S.; Miller K. L.; Clark J. H. The importance of being porous: polysaccharide-derived mesoporous materials for use in dye adsorption. RSC Adv. 2012, 2, 8992–8997. 10.1039/c2ra21367b.

[ref18] Shuttleworth P. S.; Parker J.; Budarin V. L.; Breeden S. W.; Macquarrie D. J.; Luque R. L.; White R.; Clark J. H. Starbon: Preparation, applications and transition from laboratory curiosity to scalable product. NSTI Nanotechnol. 2011, 4, 766–769.

[ref19] Milescu R. A.; Dennis M. R.; McElroy C. R.; Macquarrie D. J.; Matharu A. S.; Smith M. W.; Clark J. H.; Budarin V. L. The role of surface functionality of sustainable mesoporous materials Starbon on the adsorption of toxic ammonia and sulphur gasses. Sustainable Chem. Pharm. 2020, 15, 100230–100239. 10.1016/j.scp.2020.100230.

[ref20] Kim S.; Escamilla-Pérez A. M.; De bruyn M.; Alauzun J. G.; Louvain N.; Brun N.; Macquarrie D.; Stievano L.; Stievano L.; Boury B.; Boury B.; Monconduit L.; Monconduit L.; Mutin P. H. Sustainable polysaccharide-derived mesoporous carbons (Starbon) as additives in lithium-ion batteries negative electrodes. J. Mater. Chem. A 2017, 5, 24380–24387. 10.1039/C7TA08165K.

[ref21] Ho C.; Yu J. C.; Kwong T. A.; Mak C.; Lai S. Morphology-Controllable Synthesis of Mesoporous CeO_2_ Nano- and Microstructures. Chem. Mater. 2005, 17, 4514–4522. 10.1021/cm0507967.

[ref22] Matharu A. S.; Ahmed S.; Almonthery B.; Macquarrie D. J.; Lee Y. S.; Kim Y. Starbon/High-Amylose Corn Starch-Supported N-Heterocyclic Carbene–Iron(III) Catalyst for Conversion of Fructose into 5-Hydroxymethylfurfural. ChemSusChem 2018, 11, 716–725. 10.1002/cssc.201702207. 29281175

[ref23] García A. M.; Hunt A. J.; Budarin V. L.; Parker H. L.; Shuttleworth P. S.; Ellis G. J.; Clark J. H. Starch-derived carbonaceous mesoporous materials (Starbon) for the selective adsorption and recovery of critical metals. Green Chem. 2015, 17, 2146–2149. 10.1039/C5GC00154D.

[ref24] Suresh R.; Ponnuswamy V.; Mariappan R. Effect of annealing temperature on the microstructural, optical and electrical properties of CeO_2_ nanoparticles by chemical precipitation method. Appl. Surf. Sci. 2013, 273, 457–464. 10.1016/j.apsusc.2013.02.062.

[ref25] Ruckman M. W.; Chen J.; Qiu S. L.; Kuiper P.; Strongin M.; Dunlap B. I. Interpreting the near edges of O_2_ and O_2_- in alkali-metal superoxides. Phys. Rev. Lett. 1991, 67, 2533–2566. 10.1103/PhysRevLett.67.2533. 10044450

[ref26] Groen J. C.; Peffer L. A. A.; Pérez-Rami′rez J. Pore size determination in modified micro- and mesoporous materials. Pitfalls and limitations in gas adsorption data analysis. Microporous Mesoporous Mater. 2003, 60, 1–17. 10.1016/S1387-1811(03)00339-1.

[ref27] Liu C. J.; Wang G. X.; Sang S. X.; Rudolph V. Experimental study of supercritical CO_2_ -H_2_O-coal interactions and the effect on coal permeability. Fuel 2010, 89, 2665–2672. 10.1016/j.fuel.2010.03.032.

[ref28] Dauscher A.; Hilaire L.; LeNormand F.; Muller W.; Maire G.; Vasquez Characterization by XPS and XAS of supported Pt/TiO_2_/CeO_2_ catalysts. Surf. Interface Anal. 1990, 16, 341–346. 10.1002/sia.740160173.

[ref29] Paparazzo E.; Ingo G. M.; Zacchetti N. J. X-ray induced reduction effects at CeO_2_ surfaces: An x-ray photoelectron spectroscopy study. J. Vac. Sci. Technol. A 1991, 9, 1416–1420. 10.1116/1.577638.

[ref30] Böcklein S.; Günther S.; Wintterlin J. High-Pressure scanning tunneling microscopy of a silver surface during catalytic formation of ethylene oxide. Angew. Chem., Int. Ed. 2013, 52, 5518–5521. 10.1002/anie.201210209. 23592599

[ref31] Eloirdi R.; Cakir P.; Huber F.; Seibert A.; Konings R.; Gouder T. X-ray photoelectron spectroscopy study of the reduction and oxidation of uranium and cerium single oxide compared to (U-Ce) mixed oxide films. Appl. Surf. Sci. 2018, 457, 566–571. 10.1016/j.apsusc.2018.06.148.

[ref32] Zhou J. H.; Sui Z. J.; Zhu J.; Li P.; Chen D.; Dai Y. C.; Yuan W. K. Characterization of surface oxygen complexes on carbon nanofibers by TPD, XPS and FT-IR. Carbon 2007, 45, 785–796. 10.1016/j.carbon.2006.11.019.

[ref33] Patil A. J.; Kumar R. K.; Barron N. J.; Mann S. Cerium oxide nanoparticle-mediated self-assembly of hybrid supramolecular hydrogels. Chem. Commun. 2012, 48, 7934–7936. 10.1039/c2cc33351a. 22763813

[ref34] Tan Z.; Wu T. S.; Soo Y. L.; Peng Y. K. Unravelling the true active site for CeO_2_-catalyzed dephosphorylation. Appl. Catal., B 2020, 264, 118508 10.1016/j.apcatb.2019.118508.

