# Supplementary material for: Highly Efficient Mesoporous Carbonaceous CeO2 Catalyst for Dephosphorylation
Source: ACS Omega. 2022 Jun 17;7(26):22551–8. doi: 10.1021/acsomega.2c01832 (PMC9260935; doi:10.1021/acsomega.2c01832)
Supplement: Supplementary file 1 — ao2c01832_si_001.pdf [file ao2c01832_si_001.pdf]

Supplementary

Highly Efficient Mesoporous Carbonaceous CeO_2 catalyst for Dephosphorylation

Aashima Sharma [a,b], Surinder K. Mehta,[b] and Avtar S. Matharu[a*]

a. *Green Chemistry Centre of Excellence, Department of Chemistry, University of York, England YO10 5DD.*

b. *Department of Chemistry and Centre for Advanced Studies in Chemistry, Panjab University, Chandigarh, India 160014.*

Characterization

FTIR Data were obtained using PerkinElmer FTIR Spectrum 400 Analyser. Specific surface areas and pore size distributions were determined by BET (Braunauer-Emmett-Teller) and BJH (Barrett-Joyner-Halenda) methods. The pretreatment was carried out 150ºC to clean the surface of the synthesized material for 10 hr. Nitrogen adsorption-desorption isotherms were measured at -196°C using Micromeritics ASAP 2010 porosimeter, applying nitrogen molecule as the adsorbate. Scanning Electron Microscope (SEM) images were taken using JEOL JSM-6500F instrument operated at an accelerating voltage of 200 kV. High Resolution Transmission Electron Microscope (HR-TEM) images were taken using JEOL 1000 with an accelerating voltage of 200kV. Powder X-Ray diffraction (XRD) patterns were recorded using a PANalytical X'Pert Pro MPD diffractormeter with CuKα radiation (λ = 1.54056 Å) in the 10°-45° 2θ range. A Kratos Axis Ultra DLD system was used to collect XPS spectra using monochromatic Al Kα X-ray source operating at 120 W (10 mA x 12 kV). Data was collected with pass energies of 160 eV for survey spectra, and 20 eV for the high-resolution scans with step sizes of 1 eV and 0.1 eV, respectively. Thermogravimetric analysis (TGA) and Differential Scanning Calorimetery (DSC) was done STA 625 thermal analyser. Samples were mounted in an alumina crucible (sample weight <15.0 mg) and heated at 10°C min-1 to 600°C under flowing nitrogen gas (50 mL/min). Solid state 13C CPMAS Spectra were acquired using a 400 MHZ Bruker Advance III HD spectrometer. Chemical shifts were reported with respect to TMS and were referenced using Admantane (29.5 ppm) as an external secondary reference. ICP-MS elemental analysis was conducted at Yarra Laboratory in York (UK).

Mechanism of formation of CeO_2 decorated Starbon

The fabrication of Starbon and metal decorated Starbon involves three major steps: Expansion, Freeze drying and pyrolysis [1]. The strategy is to develop a mesoporous framework with metal oxide embedded into the network. First, the expansion of Hylon VII using a greener and sustainable approach, microwave method was employed. It is basically a process of gelatinization in water that commences the disorders the dense biopolymer network of amylose rich Hylon VII [1]. After this initiation step, the second step is retrogradation which involves partially

recrystallization process. The retrogradation process at low temperature for 48 h disintegrates the crystalline structure of amylose molecules and converts into a viscous solution [2]. In the end, the gel is macerated and exchange of solvent with lower-surface tension solvent is done which prevents collapse of network structure during drying process. During the drying process, the expanded mesoporous starch is formed. The pyrolysis step, fast carbonization and fixing of the mesoporous structure ensues. The pyrolysis step is carried out at 400°C. The temperature has a dominant role in the formation of mesoporous structure. The temperature above 300°C, a conjugated system is formed which rearranges itself into 2D aromatic planar system [3]. The system was labelled as Starbon@400.

The polysaccharides are hugely diversified in terms of structure and functionality, and those not requiring purification are among the "greenest" possible materials. CeO_2 is high in demand for their redox properties that can be being substantially altered by the porosity, crystallinity, morphology, doping, particle size and, last but not least, their arrangement in hierarchical structures from the macro- to the nano- and micro-scale. CeO_2 were synthesized at the second step during solvent exchange process. During this sodium hydroxide was added, the role of the base is to provide hydroxyl ions which are available to Ce^{3+} ions to form cerium hydroxide. During pyrolysis, the hydroxide is converted to oxide form. The morphology and size depend the heating rate and temperature of pyrolysis. The sample was labelled as CeO_2 S-400.

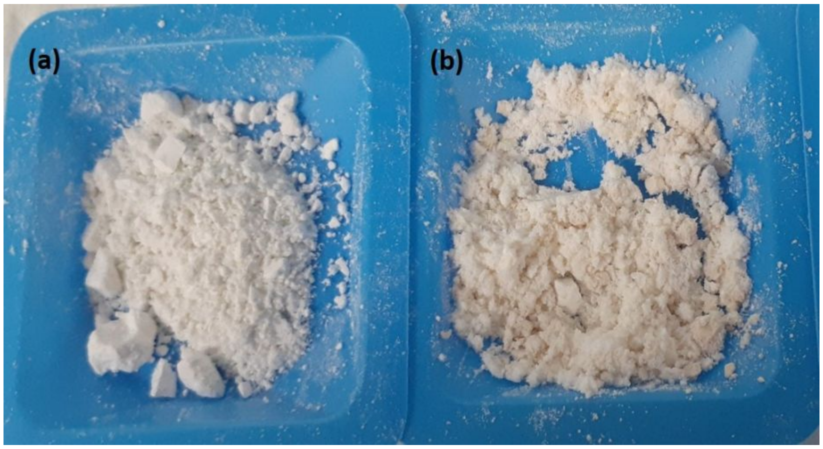

Figure S1. Picture obtained after synthesis of (a) Expt and (b) Ce-Expt

X-ray Diffraction (XRD) analysis

The XRD patterns of Starbon@400 and CeO_2-S400 are shown in Figure S2. A broad and weak diffraction peak which is centered around 20° (Figure S2a) indicates the amorphous nature of Starbon@400 [1]. The is presence of some weak i.e. masked peaks at 2θ = 28.5°, 33.0° which correspond to the (111) and (200) which implies CeO_2 fluorite phase (Figure S2b). There is presence of aluminum peaks in the XRD pattern that is due to the sample holder which absorbs X-ray radiations more strongly [4]. Therefore, XRD reveals the amorphous nature and indication of existence of CeO_2 in the carbonaceous network.

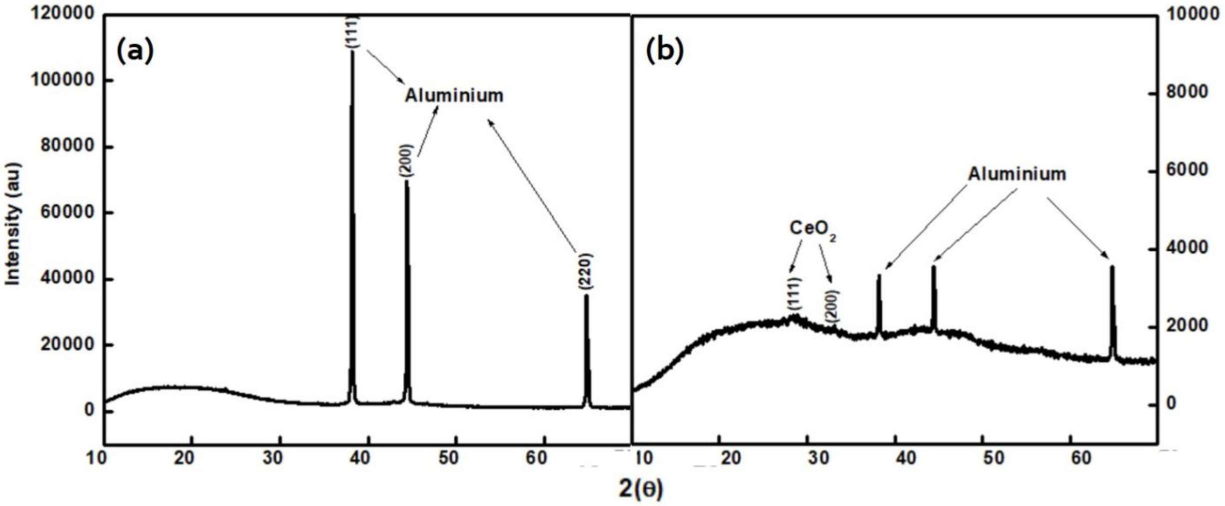

Figure S1. XRD analysis of (a) Starbon@400 (b) CeO_2-S400

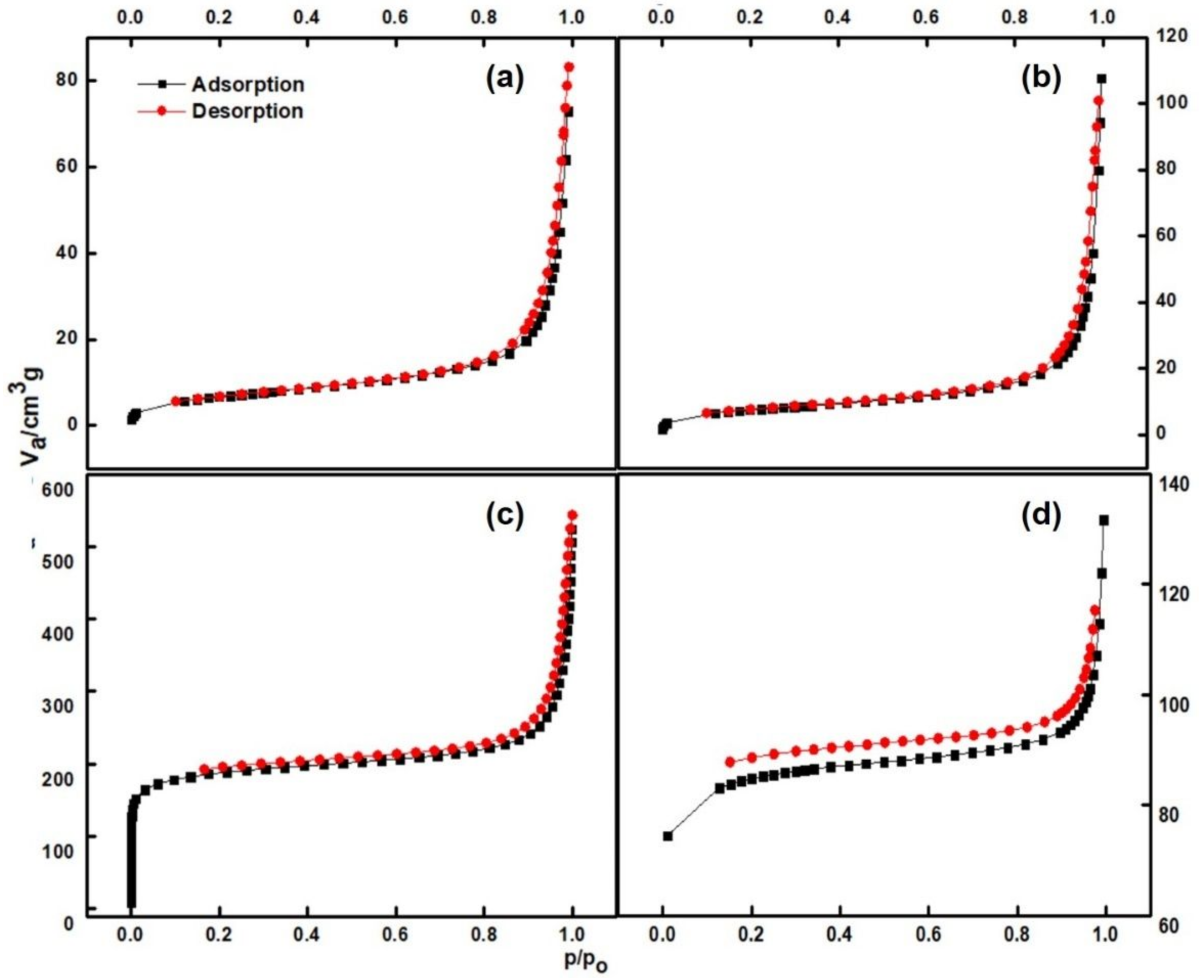

Figure S3. BET Curve (a)Expt Starch (b) CeO_2 decorated Expt Starch (c) Starbon@400 (d) CeO_2 decorated Starbon@400

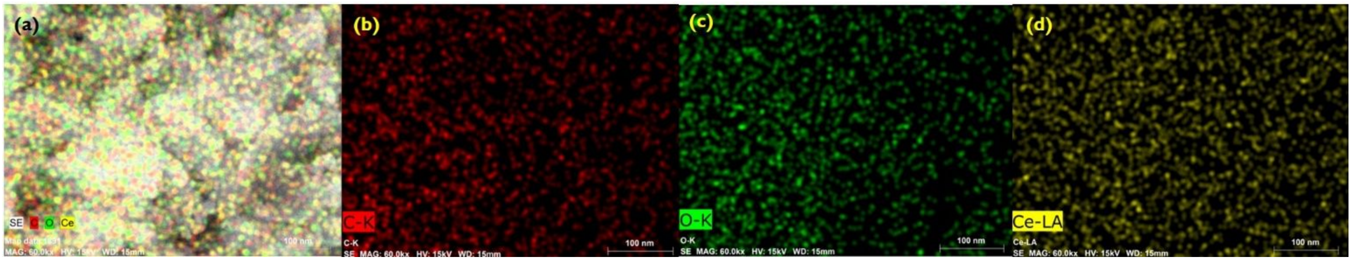

Figure S4. EDX mapping of (a) CeO_2 decorated Starbon@400 (b) Carbon (c) Oxygen (d) Cerium

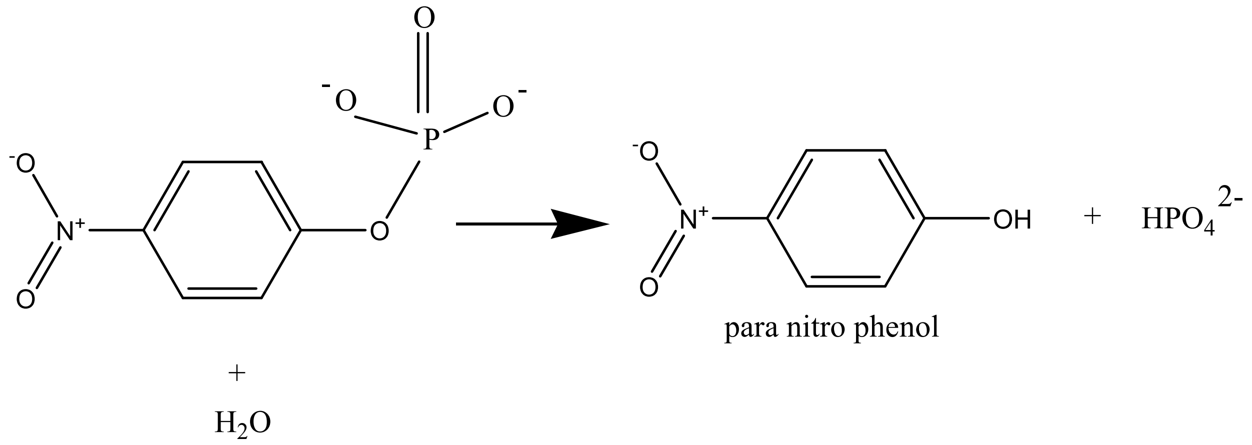

Figure S5. Schematic representation of dephosphorylation reaction

According to the Beer−Lambert law, the light absorption of the solution is positively related with the concentrations of solutes in a definite concentration range. Calibration curves were obtained from the standard solutions. The amounts of reactants and products can be quantificationally derived from the calibration curves (Figure S4).

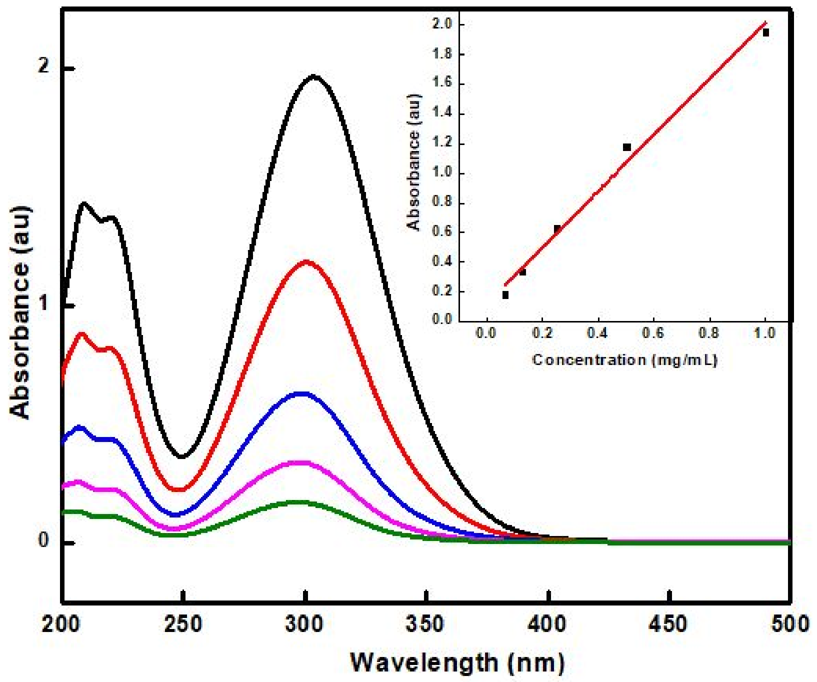

*Figure S6. Calibration graph for p-*NPP

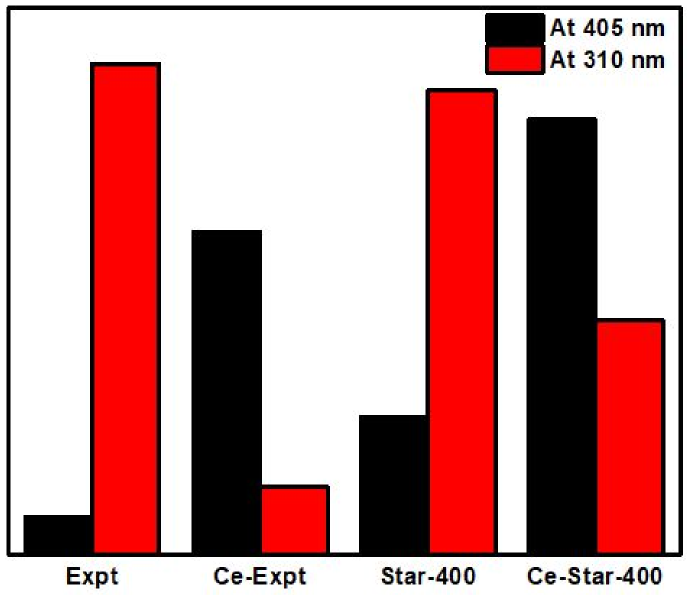

Figure S7. Comparison of prepared samples for dephosphorylation reaction

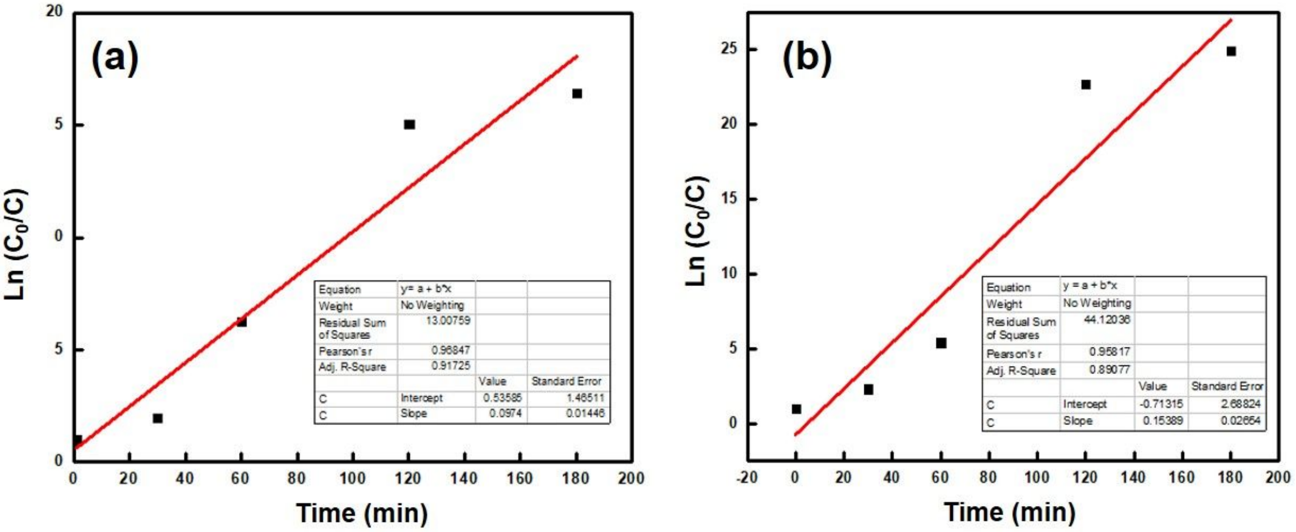

Figure S8. First order kinetic fitting of (a) CeO_2-ES and (b) CeO_2-S400, respectively.

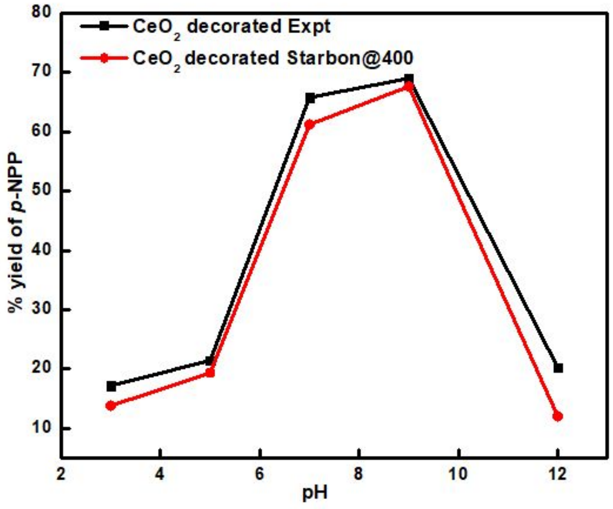

Figure S9. Effect of pH on the catalytic activity of prepared samples

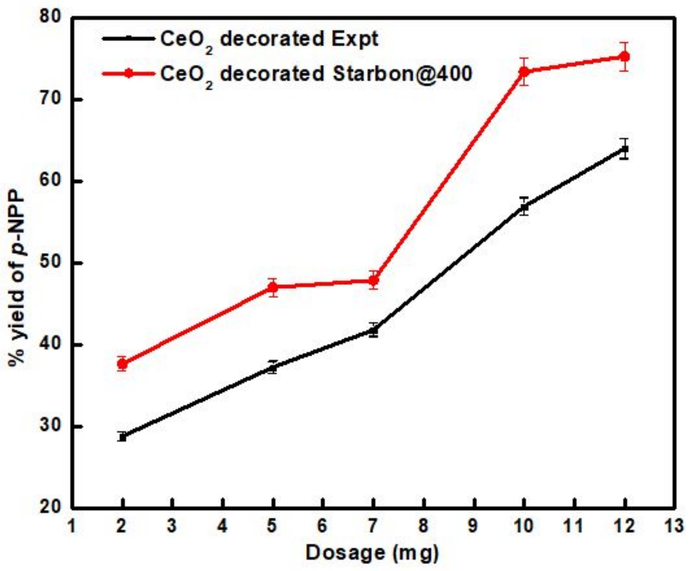

Figure S10. Effect of\dosage of catalyst

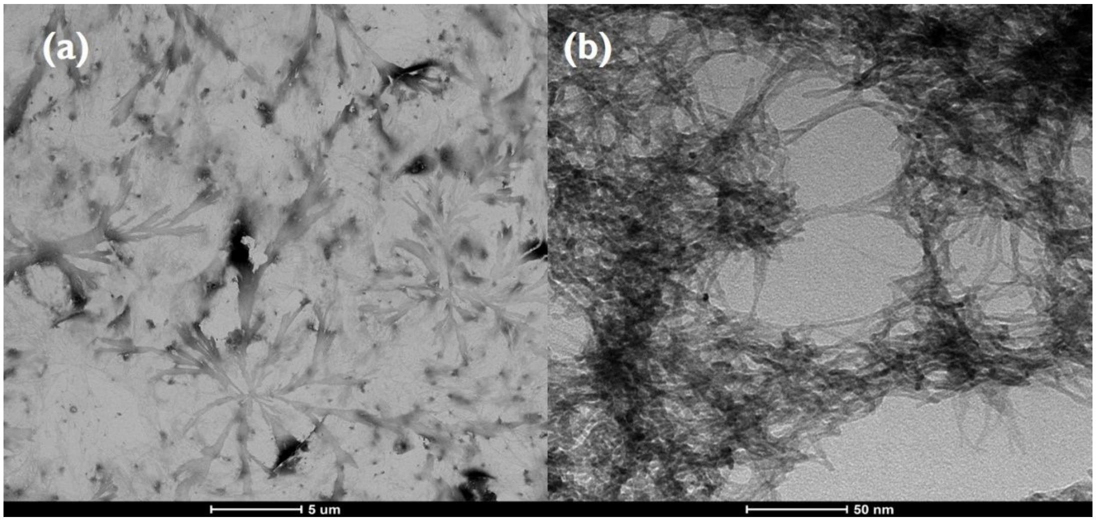

Figure S11. TEM of (a) CeO_2 decorated Expt and (b) CeO_2 decorated Starbon@400 after catalysis, respectively.

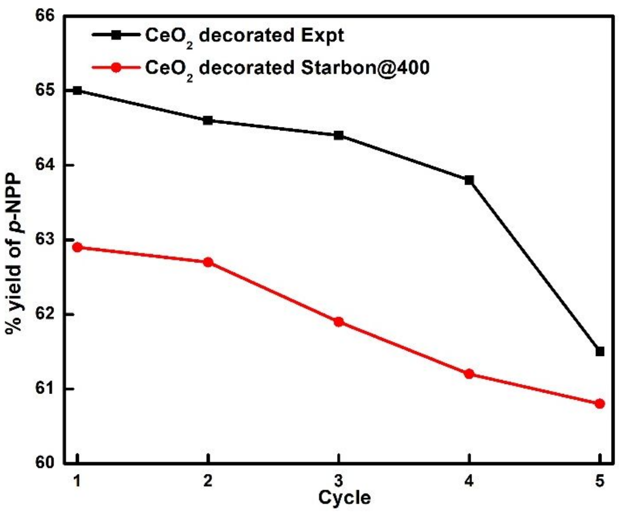

Figure S12. Recyclability of the systems

References

1. Budarin, V.; Clark, J. H.; Hardy, J. J. E.; Luque, R.; Milkowski, K.; Tavener S. J.; Wilson, A. Starbons: New starch-derived mesoporous carbonaceous materials with tunable properties. J. *Angew. Chem. Int. Ed.*, **2006**, *45*, 3782–3786.
2. Zhang, Z.; Macquarrie, D. J.; Clark, J. H.; Matharu, A. S. Chemical modification of starch and the application of expanded starch and its esters in hot melt adhesive. *RSC Adv*., **2014**, *4*, 41947–41955.
3. Zuin, V. G.; Budarin, V. L.; Bruyn, M. D.; Shuttleworth, P. S.; Hunt, A. J.; Pluciennik, C.; Borisova, A.; Dodson, J.; Parker, H. L.; Clark, J. H. Polysaccharide-derived mesoporous materials (Starbon®) for sustainable separation of complex mixtures. *Faraday Discuss.*, **2017**, *202*, 451–464.
4. Maleki, A.; Taherizadeh, A.R.; Issa, H.K.; Niroumand, B.; Allafchian, A.R.; Ghaei, A. Development of a new magnetic aluminum matrix nanocomposite. *Ceramics International*, **2018**, *44 (13)*, 15079-15085.
